# Solution for the External Contour Changes in Cone Beam Computed Tomography-Guided On-demand Online Adaptive Radiotherapy for a Patient With Very Advanced Head and Neck Cancer: A Technical Case Report

**DOI:** 10.7759/cureus.67804

**Published:** 2024-08-26

**Authors:** Jinyuan Wang, Xiangkun Dai, Baolin Qu, Changxin Yan, Yuhan Kou, Xiaoyu Liu, Xiaoshen Wang, Boning Cai

**Affiliations:** 1 Department of Radiotherapy, The First Medical Center of the Chinese PLA General Hospital, Beijing, CHN; 2 Clinical Application Training Department, Varian Medical System, Beijing, CHN

**Keywords:** on-demand art, external contour changing, head and neck tumors and diseases, online adaptive radiotherapy, image-guided radiotherapy

## Abstract

This article presents a case of a patient with advanced head and neck cancer, characterized by a large and protruding tumor. The patient was treated with an innovative on-demand online adaptive radiotherapy (ART) technology, guided by cone beam computed tomography (CBCT), on the Ethos adaptive radiotherapy platform (version 1.0, Varian Medical Systems, Palo Alto, CA). A solution was provided for this special case to address the issue where part of the target volume could not participate in the optimization due to exceeding the external contour boundary during online adaptive radiotherapy. The treatment outcome was satisfactory in terms of tumor regression, while only grade 1 radiodermatitis and grade 2 oral mucositis at the end of radiotherapy. This article discusses the clinical diagnosis, treatment process, and follow-up of this case, aiming to provide clinical references for a broader application of this technology.

## Introduction

With the rapid development of artificial intelligence and computer technologies and their extensive application in the medical field, radiotherapy - one of the pillars of cancer treatment - has also undergone significant technological development. Online adaptive radiotherapy (OART), which is one of the latest advancements in radiotherapy, is gradually altering the landscape of tumor treatment and demonstrating remarkable dosimetric advantages in the treatment of head and neck cancers. 

In the 1990s, Yan et al. proposed the concept of adaptive radiation therapy, defining it as a closed-loop radiation treatment process where the treatment plan can be modified using systematic feedback of measurements. This approach aims to improve radiation treatment by monitoring different variations and then re-optimizing the treatment plan during the course of the treatment [1]. Due to the numerous organs at risk (OARs) in head and neck cancer and long treatment courses, most patients experience shrinkage of the target volume or OARs during the treatment course. Therefore, the traditional single treatment plan approach may lead to excessive radiation dose to OARs along with the treatment course progress, aggravating the toxic side effects of radiotherapy. Adaptive radiotherapy, which adjusts dose distribution according to changes in between fractions, becomes desirable to improve clinical outcomes. Online ART demonstrates excellent prospects in both conventional radiotherapy and stereotactic radiotherapy (SRT). It can ensure adequate dose to the target volumes and has better sparing for OARs during the treatment course, especially in the head and neck region [2]. For cancers with significant daily anatomical variations in tumors and OARs, daily OART is more appropriate. In contrast, for cancers where the target volume and OARs are relatively stable throughout the radiotherapy course, with gradual changes typically occurring only one to two weeks after the treatment begins, the daily treatment adaptation might increase clinical workload without significant clinical benefits. Therefore, offline adaptive radiotherapy (offline ART) is usually preferred.

As seen in a review article by Morgan and Sher, numerous studies have explored adaptive radiotherapy for head and neck tumors, whether based on anatomical structure changes (anatomy-adapted ART, A-ART) or response (response-adapted ART, R-ART) [3]. Most of the published implementations employ offline ART, involving re-simulation, target volumes and OAR delineation, radiotherapy plan re-design, patient-specific quality assurance (PSQA), and other repetitive works. These tasks can take up to several days, and even more time is required for head and neck tumors with numerous OARs.

Ethos (version 1.0, Varian Medical Systems, Palo Alto, CA) is an intelligent and versatile online adaptive radiotherapy system based on cone beam computed tomography (CBCT) guidance. Clinicians can trigger online adaptive mode during the image-guided radiotherapy (IGRT) workflow whenever the need for adaptive is identified through two independent pre-prepared RT intents. In this case, an approximately 30-minute online adaptive treatment session and the generated adaptive radiotherapy plan were used to continue the treatment course, replacing the conventional time-consuming and labor-intensive offline adaptive workflow without treatment interruption, also significantly reducing the time and cost and improving the treatment efficiency. This process is also known as on-demand ART [4].

For very advanced head and neck cancers with a relatively large and fast-responding disease, the optimal timing to trigger treatment adaptation is when there are obvious changes in the target volume or the external contour [5]. Although the change in OARs has no advantage for clinical side effects such as xerostomia, the change of the outer contour is crucial for online adaptive radiotherapy [6]. In this paper, we report a case of a patient diagnosed with very advanced carcinoma of the floor of the mouth and treated with the on-demand ART approach guided by CBCT using the Ethos system. Additionally, we provide an online ART solution to deal with the external contour changes in such patients.

## Case presentation

Clinical presentation

The patient was a 61-year-old male with stage cT4bN3M0 carcinoma. Based on the patient's past medical history, clinical manifestations, and ancillary examinations, a definite diagnosis of carcinoma of the floor of the mouth was made. Subsequently, an intraoral approach mandibular mass biopsy was performed. Postoperative pathology showed well-differentiated squamous cell carcinoma of the mandible. Immunohistochemistry results were as follows: HER-1 (epidermal growth factor receptor (EGFR)) (+), Vimentin(-), p53(+15%), programmed death 1 (PD-1) (lymphocytes: 10%), programmed death ligand 1 (PDL1) (SP263) (CPS: 5), p16(-), HER-2 (0), and Ki-67 (+20%). The patient and his family declined concurrent chemoradiotherapy initially, so paclitaxel (albumin-bound) combined with cisplatin and fluorouracil was administered every three weeks for a total of four cycles; cetuximab 400 mg was given intravenously once a week. Subsequently, due to the disease progress, the treatment was changed to chemotherapy plus immunotherapy. The patient received chemotherapy combined with Teraplizumab every three weeks for a total of three cycles. Since there was a significant growth in the lesion, radiotherapy was initiated. After thorough communication and discussion with the patient and his family, the Ethos system was employed to provide CBCT-guided on-Demand adaptive radiotherapy with intensity-modulated radiotherapy (IMRT) technique.

Simulation

This patient was simulated in a supine position with a thermoplastic head and neck mask for immobilization and localization purposes by a Siemens SOMATOM Definition AS CT Simulator (Siemens, Hoffman Estates, IL, USA) with a selected slice thickness of 3 mm. Additionally, the patient was simulated in the same position using a United Imaging Omega MR Simulator (United Imaging, Shanghai, China), with a slice thickness of 3 mm and a T1+C sequence.

**Figure 1:**
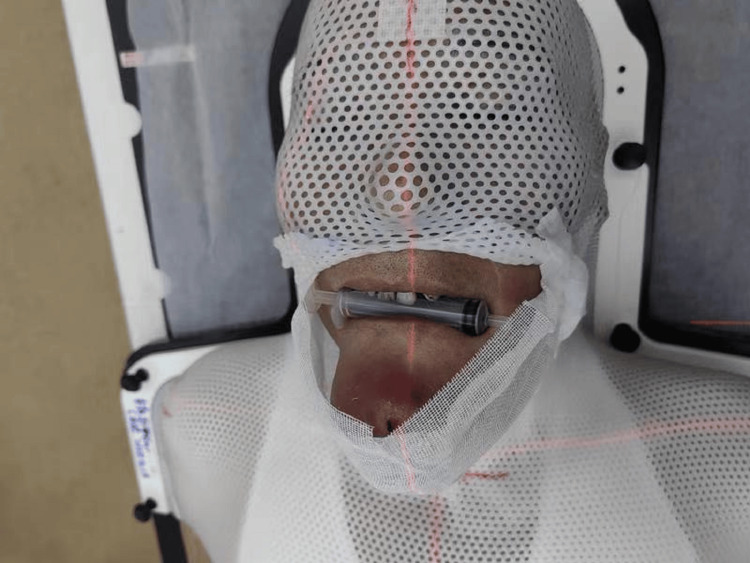
Schematic diagram of the personalized customized fixation mask for the patient

Due to the tumor invading the submental skin and having a relatively large outward-protruding volume, the mask was cut at the chin and neck to avoid extrusion and maintain fixation. The patient had difficulty opening his mouth to employ an oral stent. To reduce the irradiation of the upper wall of the oral cavity, a 10 ml syringe was held in the mouth instead. A personalized mask was customized for the patient to accommodate the unique clinical scenario, as shown in Figure 1.

Delineation, prescription, and OAR dose sparing

The simulated CT and MR images were imported into the Pinnacle (Version 10.0; FAST Multimedia, Munich, Germany) planning system for image registration workflow. The plain CT images were fused with both contrast CT and MR images, and then the target volumes and OARs were delineated according to the published National Comprehensive Cancer Network (NCCN) guidelines for head and neck cancers [7]. The planning target volume (PTV) included PTVp-H, PTVn-H, PTVpn-I, and PTVn-L. GTVp was defined as the gross tumor volume (GTV) of the primary tumor, which encompassed the tumor area of the floor of the mouth and mandible with a margin of 3 mm to PTVp-H. GTVn was defined as the GTV of metastatic lymph nodes, including the bilateral lymph node metastasis in regions Ⅰ and Ⅱ with a margin of 3 mm to PTVn-H. The prescribed doses for both PTVp-H and PTVn-H were 70 Gy in 33 fractions (2.12 Gy per fraction). CTVpn-I covered the oropharynx, floor of the mouth, and bilateral cervical lymphatic drainage areas Ⅰb, Ⅱ and Ⅲ. PTVpn-I was the expansion of CTVpn-I with a margin of 3mm, for which the prescribed dose was 60 Gy in 33 fractions (1.82 Gy per fraction). CTVn-L included the bilateral cervical lymphatic drainage area Ⅳ, expanding 3mm margin to PTVn-L with a prescribed dose of 54Gy in 33 fractions (1.64 Gy per fraction). Both the expansions of PTVpn-I and PTVn-L did not exceed 3mm under the skin. The dose constraints for various OARs are stated as follows: for bilateral parotids: D_mean _< 2800 cGy, brainstem: D_max_ < 5400 cGy, spinal cord: D_max_ < 4500 cGy, oral cavity: V_40_ < 30%; pharynx constrictors: D_mean_ < 4500 cGy, thyroid gland: D_mean_ < 3500 cGy; For bilateral mandibular bones: V_60_ < 1 cm^3^.

Initial planning

Since the optimization and dose calculation was limited within the external body contouring in Ethos platform version 1.0, which we used in this report, it was generated automatically without operator intervention during the adaptive process. Specific attention would be needed to ensure adequate coverage for structures that are close to the surface.

**Figure 2:**
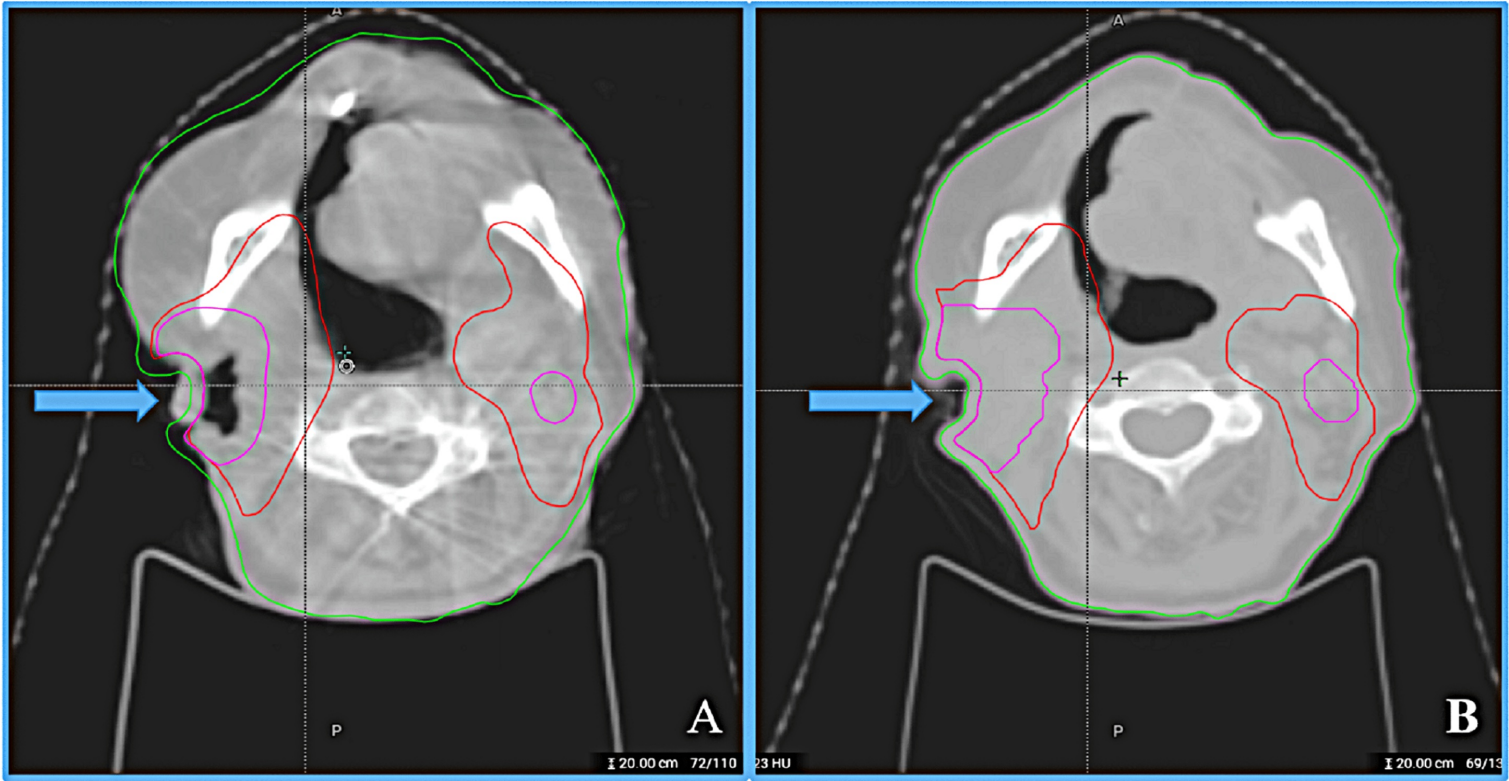
Schematic diagram of body deformation error in a patient with head and neck cancer close to the body surface The arrow in Figure A indicates that the deformed body does not completely cover the target volume in the online adaptive radiotherapy (OART) cone beam computed tomography (CBCT) and the arrow in Figure B indicates the position of the target volume and body in the simulated computed tomography (CT).

Regarding the special case of the body and handling: whether in the generation of the initial plan or the adapted plan in the online adaptive process, all plan optimizations and dose calculations are limited to within the body structure, and the parts of structures outside the body will not participate in the optimization and dose calculation. The automatic generation of the online adaptive plan is based on the synthetic CT (sCT) resampled by deforming the simulation CT to the CBCT for plan optimization and dose calculation, and the body structure of the sCT (Body_sCT) is regenerated based on the deformation vector field and cannot be modified manually. Consequently, target volumes outside the Body_sCT cannot be adjusted manually. Due to slight inaccuracy deformation of the Body_sCT during online processing, the partial target volume may exceed the Body_sCT and cannot be modified, preventing that portion of the target from participating in the optimization and causing it to receive an insufficient dose, as shown in Figure 2.

**Figure 3:**
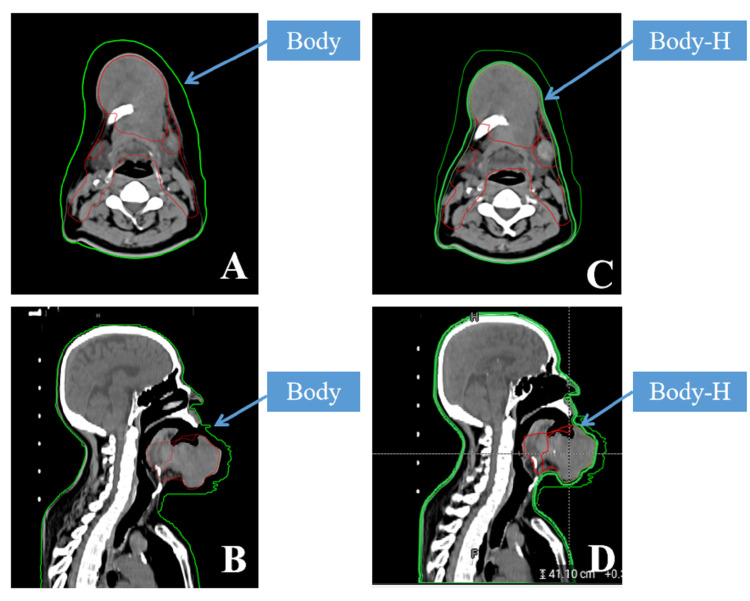
Solutions to deal with changes in the Body_sCT in the online adaptive radiotherapy (ART) process Figure A is a transverse section schematic diagram of the manual expansion of the Body area. Figure B is a sagittal plane schematic diagram of the manual expansion of the Body area. Figure C is a transverse section schematic diagram showing the relative position of Body-H and Body. Figure D is a sagittal plane schematic diagram showing the relative position of Body-H and Body.

To address this system characteristic, during the generation of the initial plan (also called reference plan) for this patient, the planner manually expanded the body contour in advance (Figure 3A, 3B); additionally, a new contour named Body-H, representing the original body structure was added (Figure 3C, 3D). During the planning process, the derivative rules for PTVpn-I, PTVp-H, PTVn-H, and PTVn-L were established in advance according to the doctor's requirements to generate the derived structures including automatic expansion of PTVpn-I, PTVp-H, PTVn-H, and PTVn-L exceeding 3mm under the skin based on Body-H. If the deformation accuracy of Body_H is not sufficient, it can be modified manually to ensure the coverage close to the body surface targets, and the target dose is not affected during the OART process avoiding the inaccuracy and inability to modify the Body_sCT. This method effectively solves the problem of inaccurate target volume near the skin by inaccurate Body_sCT in the OART process, although it may slightly impact the validation function of the manually modified parts of Body for sCT deformation accuracy on the online adapted plan.

Automatic plan generation: To ensure the plan quality, four plans were generated automatically using nine-field intensity-modulated radiation therapy (IMRT), 12-field IMRT, two full-arc volume-modulated arc therapy (VMAT), and three full-arc VMAT. All IMRT plans utilized evenly divided angles. Each plan was required to meet the clinical goals so that the prescription dose coverage of all targets reached at least 95% while the OARs received a dose as low as possible according to the dose constraints mentioned before. After comparison, the 12-field IMRT plan was chosen as the initial plan because of superior dosimetric metrics.

**Figure 4:**
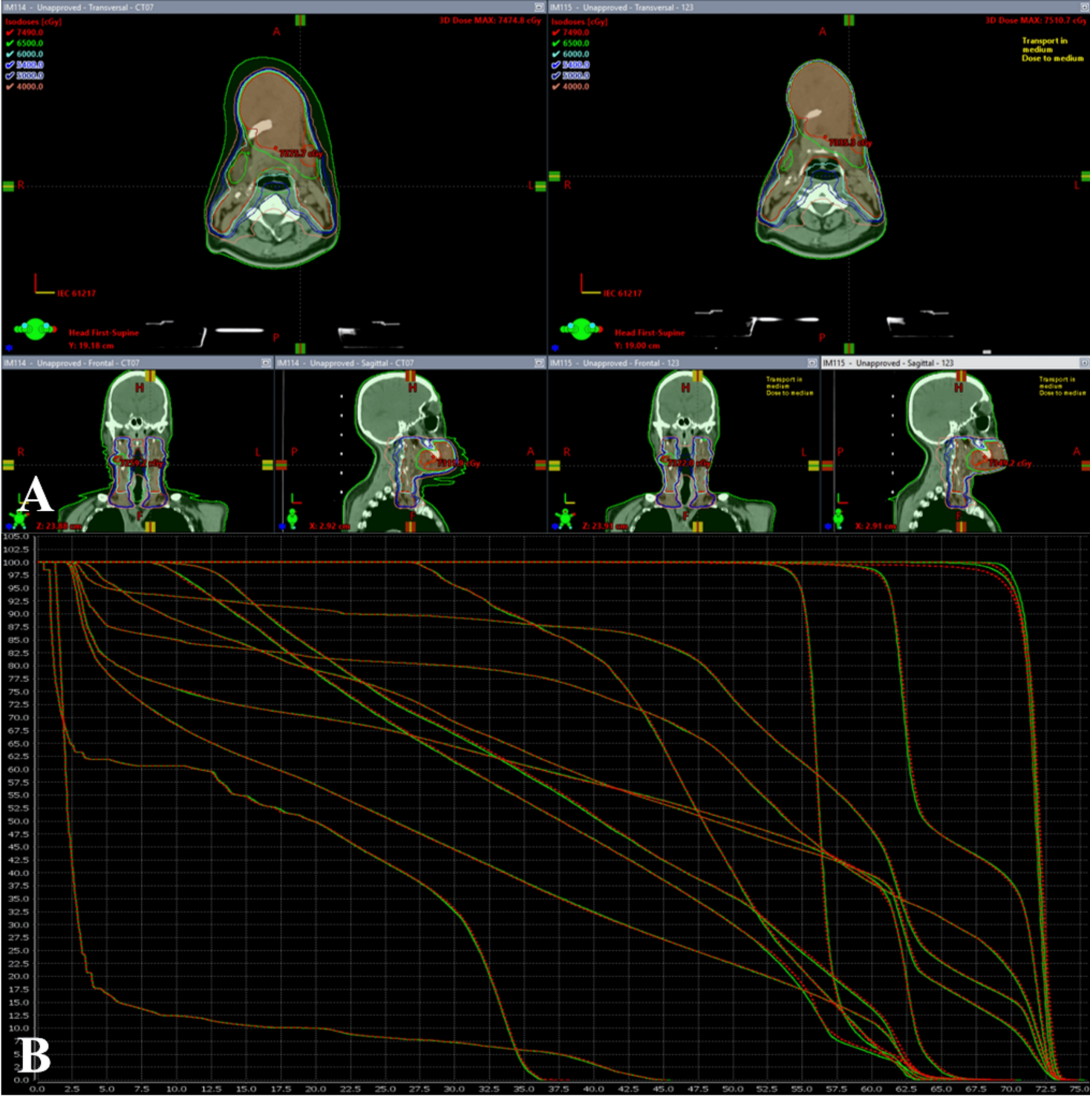
Differences after re-calculating the same plan under different body conditions Figure A shows the schematic diagram of different Body delineations and dosimetric distributions, and Figure B shows the dose-volume histogram (DVH) comparison diagram of the two Body condition plans, where the red dashed line represents the conventional skin dose result, and the green solid line represents the expanded skin dose result.

The confirmation of dose calculation accuracy for manual expansion of body: Manually expanding the body included additional air volume and reducing the surface-to-surface distance (SSD) within the calculation range to evaluate the impact of these factors on dose calculation accuracy. The initial plan was transferred to the Eclipse planning system (Varian Medical Systems, Palo Alto, CA, USA). AcurosXB dose calculation algorithm was also used to perform dose calculations with and without Body expansion. The result was compared and analyzed as shown in Figure 4. This confirmed the additional manual Body contour expansion has no significant effect on the dosimetric metrics in the initial plan (*p*>0.05).

Treatment

The patient underwent radiotherapy from April 24, 2024, to June 12, 2024, with 33 fractions in the entire treatment course, including five fractions of online adaptive sessions and 28 fractions of conventional IGRT treatment. Each fraction was monitored by an attending physician or a highly trained adaptor focusing on the changes in the target area and external contour. OART was carried out at least once every two weeks (suggested OART per week). The fractions between two OART treatments were with IGRT using the latest OART plan, that is, on-demand ART. The OART fractions occurred on the first, fifth, 10th, 20th, and 22nd treatment sessions. The process of OART using the Ethos system includes patient setting up, acquisition of initial CBCT, generation and modification of influencers, generation and modification of contours of target volumes and the OARs with clinical goals’ priorities 1 and 2, automatic plan generation (including a scheduled plan recalculated based on OART CBCT targets and OAR delineation, and an adapted plan re-optimized based on OART CBCT targets and OAR delineation), plan selection, patient-specific quality assurance (PSQA), acquisition of pre-treatment CBCT, and plan delivery. To obtain a high-quality CBCT image series during the initial CBCT scan, the patient was instructed not to swallow. When modifying the structures, in addition to adjusting the tumor area (GTVn, GTVp, CTVpn-I, CTVn-L) and OARs, the Adaptor/Doctor must also pay attention to modifying the Body-H near the target volumes. This is very important because making the Body-H close to the real skin can make the automatic derivation of the planned target more accurate. For selecting online adaptive plans, the principle of ensuring that the prescription dose coverage of all the targets reaches at least 95% while minimizing the dose received by the OARs according to the dose constraints mentioned before is followed. Plans are selected quickly by clinicians within minutes according to the dose distribution and DVH and at least not inferior to the reference plan, which can also be displayed synchronously during OART process for comparison. Among all five online ART fractions, the adapted plan was chosen four times and the scheduled plan was chosen once. The average treatment time for OART from patient setup to the end of fractionated treatment was 29.67 minutes per fraction (ranging from 24.47 to 33.17 minutes), while the average treatment time for IGRT was 5.74 minutes.

Therapeutic effect and dosimetric evaluation

**Figure 5:**
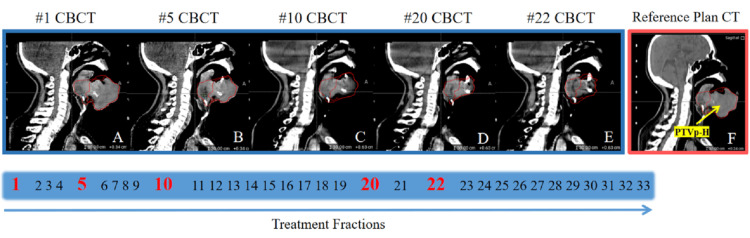
Target changes in different treatment fractions of OART in the sagittal plane Figures A to E are the sagittal plane of the same layer of cone beam computed tomography (CBCT) and target in the first to fifth OART, and Figure F is the sagittal plane of the same layer of the Sim CT target. The following is the time axis of 33 treatment fractions, in which the number of online ART fractions is displayed in bold red. OART: online adaptive radiotherapy.

At the end of the patient's treatment, the primary tumor volume had significant shrinkage (as shown in Figure 5), the side effects of radiotherapy were mild, with radiodermatitis at grade 1 and oral mucositis at grade 2. From the first OART (Fraction 1st) to the fifth OART treatment (Fraction 22nd), the volume of PTVp-H decreased from 197.20 cm^3^ to 54.66 cm^3^, a decrease of 142.54 cm^3^ (72%); the volume of PTVpn-I decreased from 610.25 cm^3^ to 387.74 cm^3^, a decrease of 222.51 cm^3^ (36%); the volume of PTVn-H decreased from 23.63 cm^3^ to 14.89 cm^3^, a decrease of 8.74 cm^3^ (37%).

In order to avoid introducing more deformation registration errors and dose accumulation inaccuracy, we compare and evaluate the scheduled, adapted, and reference plan in every five OART processes of this patient separately, rather than accumulating them together. For the target volume dosimetric metrics, along with the progress of the entire treatment course, compared to the scheduled plan, the target volume coverage rate of the adapted plan is significantly better, with the maximum difference reaching 11.45%. It is closer to the reference plan, as shown in Table 1. For other dosimetric metrics such as PTVpn-I CI and PTVp-H D_2%_, the adapted plan is also superior to the scheduled plan.

**Table 1:** Comparison of dosimetric parameters of the target volumes and OARs of the scheduled, adapted, and reference plan The CI calculation formula is: $CI=\left ( TVPV\times TVPV \right )\div \left ( TV\times PV \right )$ , where TVPV represents the volume of PTVpn-I covered by the prescription dose, TV represents the volume of PTVpn-I, and PV represents the total body volume included in the prescription dose [8]. ART: Adaptive radiotherapy; OARs: organs at risk.

		ART fraction 1	ART fraction 2	ART fraction 3	ART fraction 4	ART fraction 5	Reference Plan
PTVp-H Coverage (%Volume)	Scheduled Plan	82.94	92.56	90.01	96.05	98.88	94.62
	Adapted Plan	92.13	93.77	95.34	97.72	98.45	
PTVn-H Coverage (%Volume)	Scheduled Plan	85.27	98.21	98.73	98.1	96.44	96.76
	Adapted Plan	96.72	97.22	97.9	97.23	97.02	
PTVpn-I Coverage (%Volume)	Scheduled Plan	97.59	97.56	94.6	94.6	93.96	98.33
	Adapted Plan	98.26	98.28	98.29	98.65	97.96	
PTVn-L Coverage (%Volume)	Scheduled Plan	94.06	91.89	91.24	91.42	91.43	97.84
	Adapted Plan	97.88	98.47	98.25	97.96	98.15	
PTVpn-I CI	Scheduled Plan	0.78	0.73	0.66	0.56	0.57	0.83
	Adapted Plan	0.83	0.83	0.82	0.81	0.82	
PTVp-H D_2%_ (cGy)	Scheduled Plan	7394.42	7568.17	7601.75	7668.84	7688.86	7269.26
	Adapted Plan	7270.97	7272.37	7297.54	7329.21	7313.45	
PTVn-H D_2%_ (cGy)	Scheduled Plan	7356.13	7483.84	7535.64	7575.61	7591.46	7291.42
	Adapted Plan	7279.62	7278.98	7282.02	7274.77	7275.08	
Parotid-L D_mean_ (cGy)	Scheduled Plan	4087.7	4193.5	4237.1	3983	4198.2	3951.9
	Adapted Plan	3925.4	4101.5	3886.4	3728.6	3830.5	
Parotid-R D_mean_ (cGy)	Scheduled Plan	2919.6	2721.2	2713.1	2956.9	3071.8	2782
	Adapted Plan	2715.6	2942.5	2709.7	2970.2	3074.4	
Oral Cavity V_40_ (%Volume)	Scheduled Plan	60.04	56.76	51.46	49.36	50.89	58.1
	Adapted Plan	58.5	55.37	54.48	46.1	44.71	
Oral Cavity D_mean_ (cGy)	Scheduled Plan	4533.7	4376.9	4087.9	3970.2	4093.8	4392.5
	Adapted Plan	4465.3	4345.9	4228.7	3713	3724	
Thyroid Gland D_mean_ (cGy)	Scheduled Plan	3637.8	4020.4	3962.1	3859.1	3712.6	3650.8
	Adapted Plan	3801.7	3706	3533.8	3680.7	3507	
Pharynx constrictors D_mean_ (cGy)	Scheduled Plan	4611.6	4794.1	4872	4834.5	4807.6	4750.6
	Adapted Plan	4506.8	4325.7	4340.6	4218	4102.3	
Brainstem D_max _(cGy)	Scheduled Plan	4461.4	4546.5	4477.3	4498.4	4538.7	4479.6
	Adapted Plan	4092.4	4009.7	4200.1	4118.6	4240.9	
Spinal Cord D_max_ (cGy)	Scheduled Plan	4271.1	4155.5	4315.9	4092.7	4253.4	3768.1
	Adapted Plan	3655.4	4141.4	3807.8	3660.9	3958.7	

**Figure 6:**
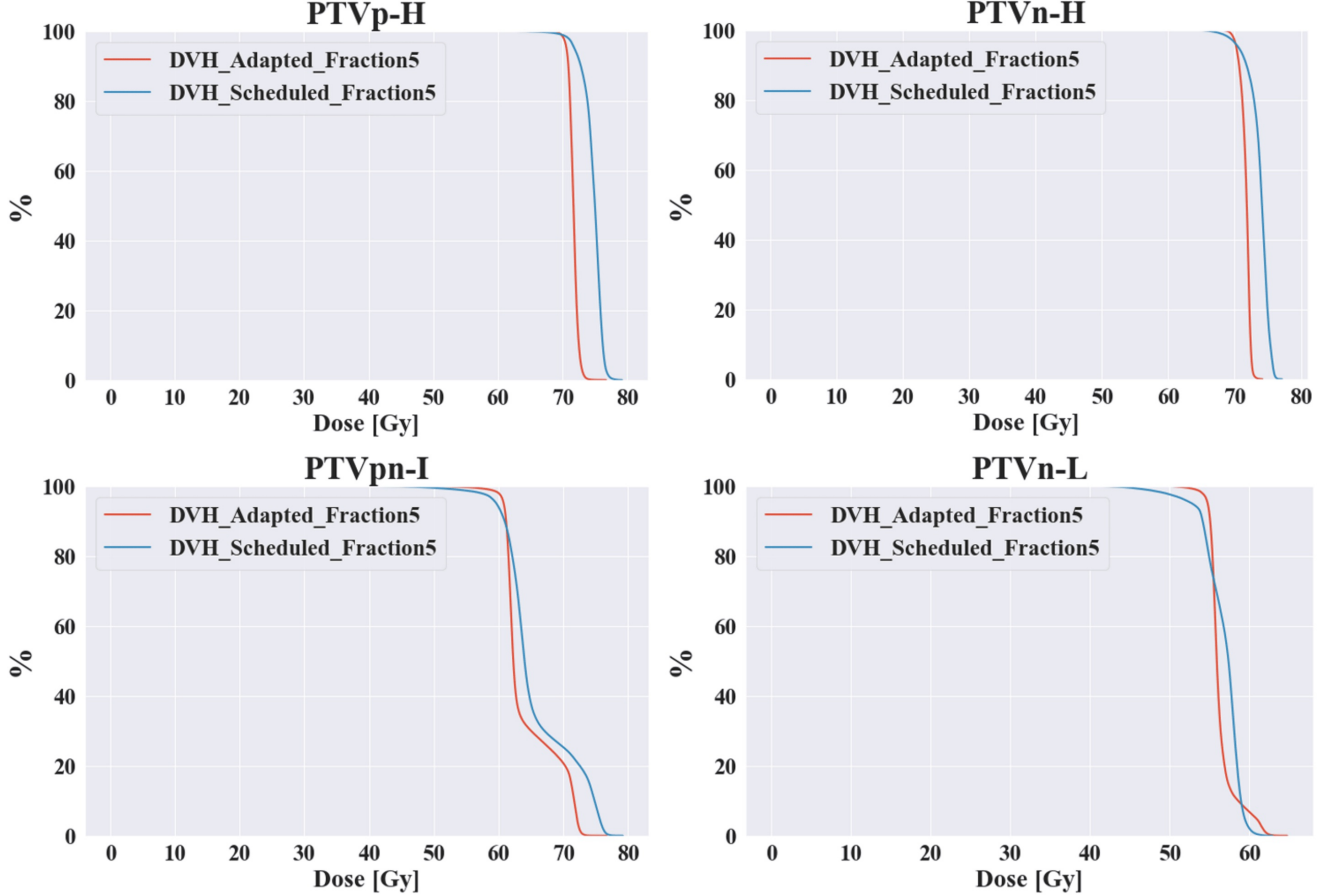
DVH differences in target volumes between the fifth adapted plan and the scheduled plan DVH: Dose-volume histogram.

**Figure 7:**
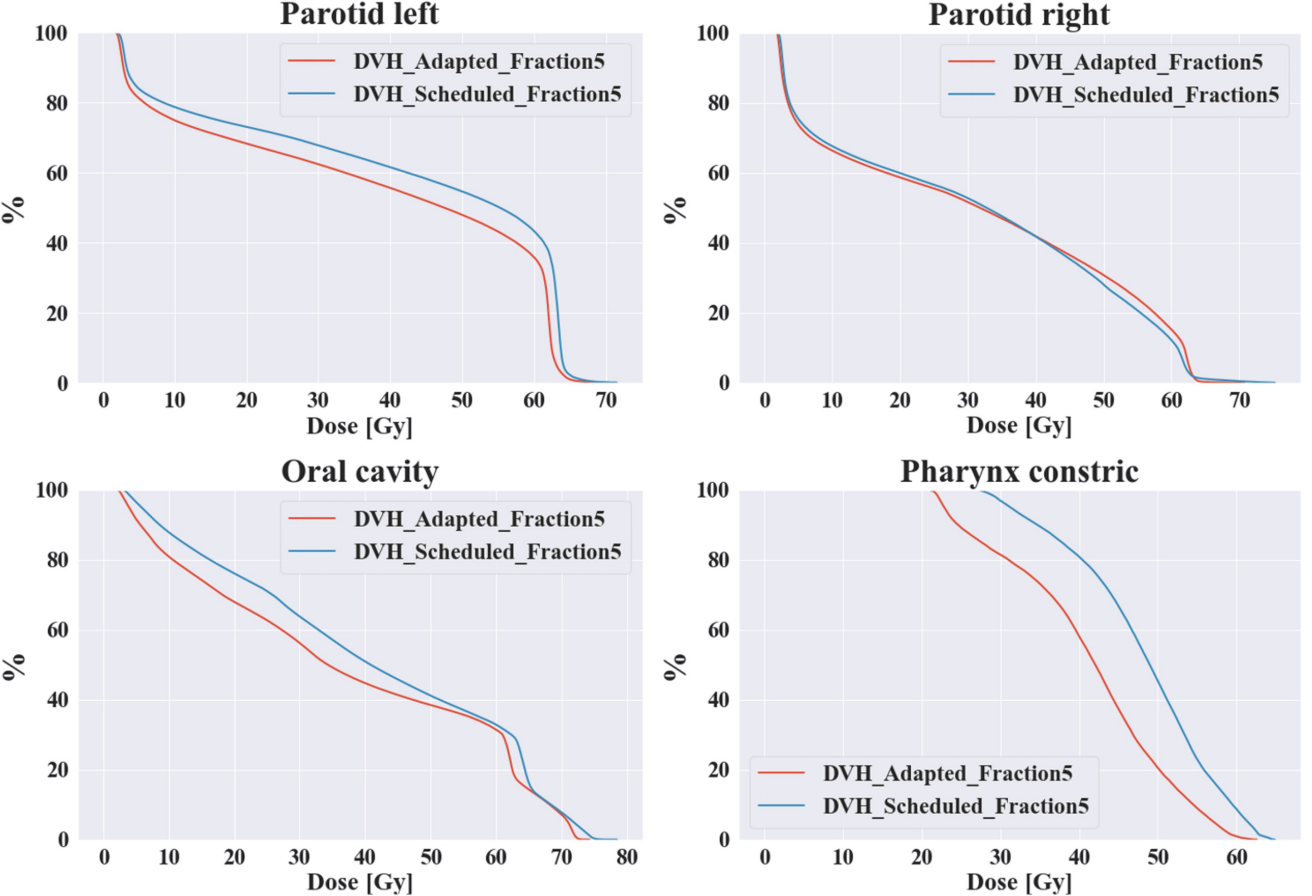
DVH differences in some OARs between the fifth adapted plan and the scheduled plan DVH: Dose-volume histogram; OARs: organs at risk.

Similarly, as can be seen from the dosimetric metrics of some OARs in Table 1, with the progress of treatment, the adapted plan has more advantages. Because as the treatment progresses, the target area has a significant shrinkage, the irradiation range of the adapted plan shrinks, and the dose of radiotherapy received by the corresponding OARs gradually shrinks. In addition, for the patient's 22nd treatment, that is, the fifth adaptive radiotherapy, we can see that there is a large difference between the adapted plan and the scheduled plan, such as the target volume coverage and the maximum dose; as well as the dose of the organs at risk, as shown in Figures 6, 7, which shows the dose-volume histogram (DVH) differences of the two planning methods of the fifth adaptive radiotherapy for different target areas and OARs.

## Discussion

Offline ART is mainly used in patients with systemic or progressive weight loss or changes in tumor morphology during the treatment course [9] and has obvious dosimetric advantages [3]. Studies by Vladimir Avkshtol et al. [10] and Philip Blumenfeld et al. [11] have found that online ART is feasible for head and neck cancer, significantly improving target prescription dose coverage and homogeneity while reducing the radiation dose to OARs. According to our case report, we highlighted the anticipation of possible changes in the tumor and the feasibility of additional workflow to ensure the accuracy of dose calculation during the ART process. Given the anticipation of these gradually changing anatomies, along with confidence in immobilization and repeatability, as well as an optimal balance between the intensity of work of attending physicians and clinical benefits, the attending physician adopted the on-demand ART approach. This is our institution's first experience with the on-demand ART approach. The current triggering conditions for on-demand ART are based on the changes in the tumor and/or external body contour monitored by our physician or adaptor. Sean All et al. [12] evaluated the potential benefits of daily online ART versus weekly online ART for the treatment of patients with head and neck squamous cell carcinoma. They found that patients with daily online ART had limited benefits, and weekly online ART significantly improved the dosimetric indicators of the target volume and OARs, which is consistent with the conclusion drawn in our case. For head and neck cancer patients, due to the relatively fixed position and anatomical location, target not changing significantly every day, and time-consuming organ delineation, using daily OART would increase unnecessary costs and patient's on-couch time. Moreover, the patient benefits would not increase compared to weekly OART.

Using the on-demand ART treatment approach can streamline the offline adaptation workflow, significantly reducing the resources needed and avoiding the potential treatment interruption in a busy clinic when compared with the conventional offline ART and daily OART. This initial experience found that treating very advanced head and neck cancers with the anticipation of significant anatomical changes using Ethos, a CBCT-based ART platform with on-demand approach is feasible and effective. It allows for generating a new plan without performing CT simulation again. However, due to the large changes in the body contour of on-demand ART patients, re-simulation is still required when there is a significant difference between sCT and CBCT under the current system design. Therefore, it is crucial to pay extra attention to the results of the deformation of sCT in clinical practice, that is, the consistency of the sCT body's external contour (Body_H) and the CBCT in this case.

The AcurosXB algorithm has satisfactory dose calculation accuracy [13,14], but manual or automatic extrapolation of the body in the initial plan can result in air and SSD changes in the calculation area. It is still recommended to import the initial plan into Eclipse for dose calculation to confirm a clinically acceptable dose calculation before treatment.

Although Ethos system version 2.0 with HyperSight can solve the problem proposed in our article that dose will be calculated directly on the HyperSight dataset without synthetic CT, because there are still many Ethos v1.x users, technological innovation is necessary to solve the issue where part of the target volume could not participate in optimization due to exceeding the external contour boundary during online adaptive radiotherapy.

## Conclusions

In this article, we described a case of a very advanced head and neck cancer patient who underwent a CBCT-guided on-demand ART treatment approach using Ethos with minimal increase of resources. The change of the patient's tumor is closely related to the change of the body's outer contour, and we provided an online ART solution to deal with the change of the body’s outer contour during the plan generation. This case highlights the benefit of this approach for managing significant outer contour changes during the online ART process. Further long-term follow-up of this patient as well as a larger cohort of patients receiving similar on-demand ART treatment approach is needed to confirm the actual clinical benefits, such as reducing high-grade toxicity and improving local control rates.
